# Development of a Video Recording and Review Process for Trauma Resuscitation Quality and Education

**DOI:** 10.5811/westjem.2018.12.40951

**Published:** 2019-02-06

**Authors:** Kathleen S. Williams, Caroline Pace, David Milia, Jeremy Juern, Jonathan Rubin

**Affiliations:** * Medical College of Wisconsin, Department of Emergency Medicine, Milwaukee, Wisconsin; † Medical College of Wisconsin, Department of Surgery, Division of Trauma and Critical Care, Milwaukee, Wisconsin

## Abstract

Video review for quality and education purposes has been a valued tool for decades. However, the use of this process dropped significantly after the development of the Health Insurance Portability and Accountability Act in the 1990s. Video review was recently reestablished at our institution. By working with our institutional legal counsel and risk management team, we have been able to create a video review process that complies with legal requirements. Literature on this subject has not described the process of obtaining video recordings. We aimed to review the process of obtaining high quality recordings in a secure manner. We hope that in the future, the data collected through our multidisciplinary review process will be helpful in improving quality of care for injured patients and providing coaching and feedback to learners, as well as improving our trauma education curriculum.

## INTRODUCTION

Video review for quality and performance improvement has been used in multiple fields since the 1960s.^1^,^2^ Previous publications have discussed the benefits of using this information for resident education and quality improvement. Video review for trauma resuscitation is particularly valuable, as patient care in this area is protocol based and involves multiple providers as part of a team. Programs using video review have been able to show improved compliance with Advanced Trauma Life Support (ATLS) guidelines.^3^ While publications are available regarding the benefits of performing video review, there is limited literature regarding development of this process. We aim to review the process of successfully reestablishing a robust video review process at our institution.

### Obstacles to Video Review

Video review for resuscitation was common in the 1980s and 1990s. A survey of trauma centers in densely populated regions of the United States (U.S.) published in 1999 revealed 20% of trauma centers were using video review, the majority of which were designated Level I trauma centers. In the study, 34% of Level I trauma centers surveyed had a video process in place for trauma resuscitation. Of the hospitals discontinuing their video review programs and those that had never used videotaping, the primary issues were medicolegal concerns and inadequate support from personnel and staff. Interestingly, surveyed hospitals actively using video review reported no medicolegal issues.^4^

The enactment into law of the Health Insurance Portability and Accountability Act (HIPAA) in 1996 discouraged many institutions from continuing this practice due to concerns regarding patient privacy and legal implications. A survey of 125 trauma centers in the U.S. in the early 2000s revealed that only 15% used a video review process, while 40% had previously had a video review process that was no longer used. The majority of these institutions reported HIPAA compliance and scarce resources as reasons for discontinuing their process.^5^

At our institution, the video review process in place in the 1990s was discontinued when a state law on voyeurism was enacted in 2001, making it illegal to video record anyone in an undressed state without prior written informed consent.^6^ This regulation did not specifically address video recording for the purpose of medical care or quality improvement. In addition, The Joint Commission and the Center for Medicare and Medicaid Services (CMS) began requiring written informed consent for video recording in healthcare in the early 2000s.^7^ Video recording and review was discontinued at our institution due to concerns regarding compliance with these regulations.

Recognizing its potential value for education and quality improvement, our institutional legal counsel and risk management developed solutions in 2015 to comply with state and federal regulations. Instrumental in this process was the language present in the Conditions of Admission (COA) form patients or their designees sign during the registration process. This document includes a section on video recording for the purpose of education and quality improvement. The consent form includes the following: “I consent to the recording, photography, closed circuit monitoring or filming for the purposes of treatment (will be in the medical record) or quality of care and teaching.” The consent is valid for one year after signing.

When a patient is critically ill and cannot sign a COA, the patient may physically or verbally sign at a later time. A family member can also sign for the patient. It is rare that a COA is not obtained. This occurs in cases such as death occurring prior to obtaining a signature, the patient is unidentified, or no family is available. This process allows us to comply with state and national regulations and limits the risk assumed by providers, as they are considered protected by our hospital quality improvement process. Along with this new COA, the risk management team assisted in developing a secure process for recording and data keeping. Integral to this process is the choice of technology and software.

## PROCESS DEVELOPMENT

### Choice of Technology

Avigilon™ in-ceiling Micro Dome cameras (Vancouver, British Columbia) were installed in three of our four trauma resuscitation bays where all of our trauma team activation patients arrive. Cameras are positioned overhead to capture care and procedures. Recordings are manually activated by a set of pushbutton switches in the center of the room. Each resuscitation bed has an individual activation button and light indicator (red or green) to indicate whether recording is live.

We initially encountered challenges developing a process to ensure video recording was activated on patient arrival. This task was assigned to our trauma technicians. In addition, we added this task to our huddle checklist performed by our physician providers prior to patient arrival. The recorded cases are stored on an isolated computer using Avigilon software. The password-protected computer is located in a locked closet with access limited to the emergency department (ED) director. In addition, this computer has no connection to the internet or campus network. Total cost for the technology and installation was approximately $9,000.

### Process of Obtaining Recordings (See Figure)

A weekly report of patients evaluated in the trauma resuscitation area is downloaded from the electronic health record (EHR). The report contains arrival time stamp, medical record number and chief complaint. The report is imported into a secured database created for case tracking and video review data collection. Cases with trauma-related chief complaints are selected for assessment of COA status in the EHR and ED disposition. In addition, cases identified through other venues are also identified as candidates for review (e.g., Trauma Process Improvement program, Trauma Surgery Case Conference, concerns or questions from individual team members). Patients with a COA on file within the prior 12 months will have the video recording computer checked for the presence of a recorded video. Recordings of non-trauma related cases or those without a COA on file are immediately destroyed prior to any review. Video recordings meeting requirements for consent are then downloaded onto an encrypted flash drive for subsequent review.

Recordings available on the computer are reviewed by either the ED director or the director of clinical operations to assess for quality. Those recordings lacking large portions of care or any sound or that have video quality issues are discarded. In addition, patients are excluded if they were discharged from the ED. Deaths occurring within the resuscitation bay are included if an active COA was on file from a previous encounter. All videos are discarded after 30 days.

### Multidisciplinary Video Review Team

A multidisciplinary video review committee was created for quality review. This team includes designated faculty members from emergency medicine, trauma surgery and anesthesia. In addition, the trauma program manager, trauma performance improvement coordinator, ED nursing leadership, and the lead ED technician are also present.

### Review Database Recording

A secure database was developed to keep records of the review process and assist with communication to the team regarding outcomes of reviews. In the database, patients are identified by a video number and medical record number. Review areas include general impression of the case and issues identified with members of the care team (faculty, resident physicians, nursing, techs, pharmacy and respiratory therapy). A section for overall learning points is provided. Finally, there is an area to list any issues identified and document a plan of action. The videos are then flagged if valuable for our monthly, multidisciplinary trauma conference. A case can also be flagged if issues arise requiring a formal quality review as part of the requirements set out by the American College of Surgeons Committee on Trauma (ACS-COT). For the purposes of education, mechanism of injury and any procedures recorded are documented.

### Use of Videos for Education and Quality Improvement

Videos are used for training, education and coaching for physician providers and nursing. Recordings are integrated into a monthly, multidisciplinary emergency medicine/trauma conference. Videos considered to be of value demonstrate effective utilization of ATLS principles, as well as show the impact of deviation from these protocols. Videos demonstrating procedural technique, leadership skills and team dynamics are used for physician coaching. The video process allows the trauma medical director to address inefficiencies or gross deviations in policy and guideline performance issues with providers in a one-on-one setting. This is used to satisfy requirements in performance improvement set forth by ACS-COT. In addition, videos are also flagged for a nursing educational archive. Nurse management uses recordings for orientation training, procedure technique improvement, and direct feedback regarding team care of an injured patient.

## CONCLUSION

Video recording for the purposes of quality improvement and education in medicine has been used for decades. The use of this process for trauma care in particular was widespread prior to the development of multiple federal and state privacy laws across the U.S. We recently began using this tool again at our institution. By working with our institution’s legal counsel, we were able to reestablish trauma video recording within the guidelines of the established privacy laws. Ongoing data collection from this process will allow our group to assess its value for the purposes of process improvement and education.

## Figures and Tables

**Figure:**
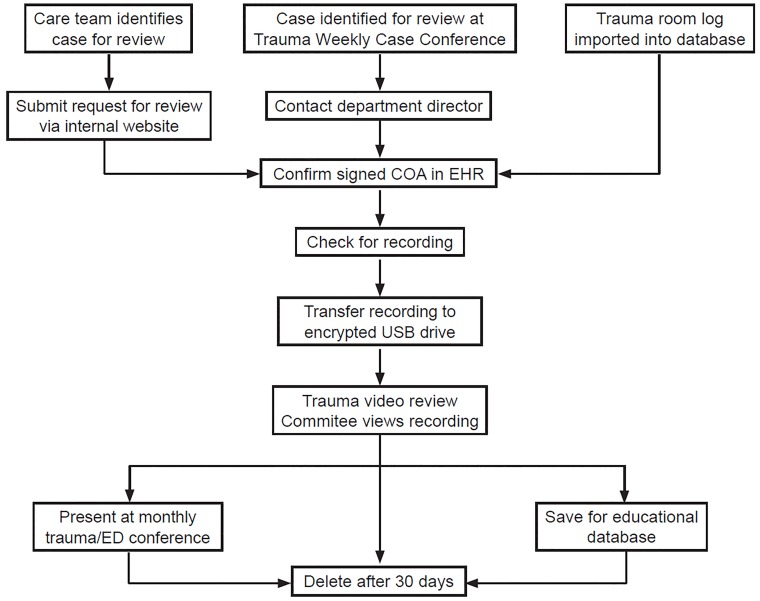
Our institutional video review process. *EHR*, electronic health record; *COA*, Conditions of Admission; *ED*, emergency department.
